# Normoxic HIF-1α Stabilization Caused by Local Inflammatory Factors and Its Consequences in Human Coronary Artery Endothelial Cells

**DOI:** 10.3390/cells11233878

**Published:** 2022-12-01

**Authors:** Mohsen Abdi Sarabi, Alireza Shiri, Mahyar Aghapour, Charlotte Reichardt, Sabine Brandt, Peter R. Mertens, Senad Medunjanin, Dunja Bruder, Ruediger C. Braun-Dullaeus, Sönke Weinert

**Affiliations:** 1 Department of Internal Medicine, Division of Cardiology and Angiology, Otto-von-Guericke University, 39120 Magdeburg, Germany; 2 Infection Immunology Group, Institute of Medical Microbiology and Hospital Hygiene, Otto-von-Guericke University, 39120 Magdeburg, Germany; 3 Clinic of Nephrology and Hypertension, Diabetes and Endocrinology, Otto-von-Guericke University, 39120 Magdeburg, Germany; 4 Immune Regulation Group, Helmholtz Centre for Infection Research, 38124 Braunschweig, Germany

**Keywords:** normoxic HIF-1α stabilization, coronary artery endothelial dysfunction, early atherosclerosis, micromilieu factors

## Abstract

**Highlights:**

- A HIF-1α biosensor was generated to facilitate studies on the role of different agents in normoxic HIF-1α stabilization.
- Micromilieu factors (MFs) induce normoxic HIF-1α stabilization with two different kinetics in HCAECs.
- There is an unknown indirect regulation of normoxic HIF-1α stabilization by NF-κB.
- Normoxic endothelial HIF-1α stabilization plays an important role in the inflammatory response of ECs to MFs, which could play a crucial role in the progression of atherosclerosis.

**Abstract:**

Knowledge about normoxic hypoxia-inducible factor (HIF)-1α stabilization is limited. We investigated normoxic HIF-1α stabilization and its consequences using live cell imaging, immunoblotting, Bio-Plex multiplex immunoassay, immunofluorescence staining, and barrier integrity assays. We demonstrate for the first time that IL-8 and M-CSF caused HIF-1α stabilization and translocation into the nucleus under normoxic conditions in both human coronary endothelial cells (HCAECs) and HIF-1α-mKate2-expressing HEK-293 cells. In line with the current literature, our data show significant normoxic HIF-1α stabilization caused by TNF-α, INF-γ, IL-1β, and IGF-I in both cell lines, as well. Treatment with a cocktail consisting of TNF-α, INF-γ, and IL-1β caused significantly stronger HIF-1α stabilization in comparison to single treatments. Interestingly, this cumulative effect was not observed during simultaneous treatment with IL-8, M-CSF, and IGF-I. Furthermore, we identified two different kinetics of HIF-1α stabilization under normoxic conditions. Our data demonstrate elevated protein levels of HIF-1α-related genes known to be involved in the development of atherosclerosis. Moreover, we demonstrate an endothelial barrier dysfunction in HCAECs upon our treatments and during normoxic HIF-1α stabilization comparable to that under hypoxia. This study expands the knowledge of normoxic HIF-1α stabilization and activation and its consequences on the endothelial secretome and barrier function. Our data imply an active role of HIF-1α in vivo in the vasculature in the absence of hypoxia.

## 1. Introduction

Cardiovascular diseases are responsible for 17.9 million deaths per year worldwide and were the main cause of morbidity and mortality, attributed to 35.3% of deaths in Germany in 2019 [1]. Atherosclerosis is the cause of various cardiovascular diseases and is characterized by initial endothelial barrier dysfunction (EBD) [2]. The hypoxia inducible factor (HIF) family is a ubiquitously expressed transcription factor family consisting of three α-subunits and one β-subunit. The α- and β-subunits are able to pair to function as effective heterodimeric transcription factors. Under normoxic conditions, in an oxygen-, iron- and 2-oxoglutarate-dependent reaction, HIF-1α is hydroxylated and thereby is marked for degradation via the von Hippel–Lindau protein and proteasomal degradation. Under hypoxic conditions, hydroxylation of HIF-1α is inhibited, preventing proteasomal degradation of the α-subunit. Stabilized HIF-1α is translocated into the cell nucleus, forming a heterodimeric complex in interaction with the HIF-1β subunit. This heterodimeric complex binds to hypoxia-response elements, which leads to adaptation of the cell metabolism to hypoxic conditions [3]. Stabilization of HIF-1α plays a crucial role in the development and progression of atherosclerotic plaque through cell-specific responses on endothelial cells (ECs), vascular smooth muscle cells (VSMCs), and macrophages as well as through promotion of monocyte recruitment [4,5,6,7]. In addition to the paramount role of HIF-1α under hypoxia, the stabilization of HIF-1α was astonishingly discovered via soluble factors under normoxic conditions [8,9,10].

Coronary arteries supply blood to the heart muscle. The development of atherosclerosis in coronary arteries leads to dramatic consequences such as heart attack or heart failure. We hypothesized that factors locally present in the growing atherosclerotic plaque (so-called micromilieu factors, MFs) induce HIF-1α stabilization in the absence of hypoxia (normoxic HIF-1α stabilization), thereby leading to an alteration of the endothelial secretome and loss of human coronary endothelial cells (HCAECs) function, resulting in their phenotypic shift toward a proatherogenic state.

## 2. Methods

### 2.1. Cell Culture

Immortalized HCAECs (Applied Biological Material, Richmond, BC, Canada) were cultivated using Endopan 3 medium (PAN-Biotech, Aidenbach, Germany) on gelatin-coated plates. HIF-1α-mKate2-expressing human embryonic kidney 293 (HEK-293) cells as well as wild-type HEK-293 cells (DSMZ, Braunschweig, Germany) were grown in Dulbecco’s Modified Eagle Medium (DMEM) with high glucose (Thermo Fisher Scientific, Waltham, MA, USA) supplemented with 10% (*v*/*v*) fetal bovine serum (FBS, Thermo Fisher Scientific, Waltham, MA, USA) and 1% (*v*/*v*) Anti-Anti (Thermo Fisher Scientific, Waltham, MA, USA). Both HCAECs and HIF-1α-mKate2-expressing HEK-293 cells were incubated at 37 °C with 5% CO_2_ and were utilized from passages 5–10 for all experiments.

### 2.2. Generation of the HIF-1α Biosensor

After the preparation of the donor vector (see Supplementary Methods), HEK-293 cells were cotransfected with the donor and the two TALE-Nuclease vectors by the calcium phosphate method as described previously [11]. Knock-in HEK-293 cells were then selected using DMEM containing hygromycin B (AppliChem, Darmstadt, Germany). To obtain a uniform HIF-1α-mKate2-expressing HEK-293 cell line, we carried out single-cell clone assays. Briefly, during cell passaging, the medium containing knock-in cells was subjected to serial dilution until there was only one cell in each well of the 96-well plate. After these single cells were grown, single cell clones were further cultured in 12-well and 6-well plates as well as in 100 × 20 mm culture dishes. Stable genomic integration into the AAVS1 locus on chromosome 19 was confirmed by junction PCR (see Supplementary Methods and Supplementary Figure S1). Finally, the uniform HIF-1α-mKate2-expressing HEK-293 cells were frozen and stored at −150 °C.

### 2.3. Live Cell Imaging

For the live cell imaging experiments, HIF-1α-mKate2-expressing HEK-293 cells were first cultivated for two days in 24-well imaging plates (Zell-Kontakt, Nörten-Hardenberg, Germany) with DMEM. After two days, the cell culture medium was changed to a medium containing Hoechst 33342 DNA stain (Thermo Fisher Scientific, Waltham, MA, USA) as well as 50 ng/mL each of tumor necrosis factor (TNF)-α, interferon (INF)-γ, interleukin (IL)-1β, IL-8, macrophage colony-stimulating factor (M-CSF), insulin-like growth factor-I (IGF-I), cocktail 1 (TNF-α, INF-γ, and IL-1β), and cocktail 2 (IL-8, M-CSF, and IGF-I). Then, we monitored HIF-1α stabilization and its localization into the nucleus for 24 h using a Zeiss Axiovert 200 m fluorescence microscope equipped with an Rolera EM-C^2^ camera (Q imaging, Tucson, AZ, USA), a cage incubator (Okolab, Ottaviano, Italy), and the appropriate filter sets.

### 2.4. Immunoblotting

HCAECs were cultivated in 145 × 20 mm culture dishes until they reached 70–80% confluency. The cells were then incubated with the cell culture medium (control group) or treated with medium containing 50 ng/mL each of TNF-α, INF-γ, IL-1β, and cocktail 1 (TNF-α, INF-γ, and IL-1β) for 12 h as well as with medium containing 50 ng/mL each of IL-8, M-CSF, IGF-I, and cocktail 2 (IL-8, M-CSF, and IGF-I) for 18 h under normoxic conditions. Next, the total cell lysates were isolated from HCAECs using a commercial kit (Active Motif, Carlsbad, CA, USA) and stored at −80 °C until further use. The protein concentration was quantified in a detergent-compatible protein assay (Bio-Rad, Hercules, CA, USA). Samples (15 μg of total protein) were separated by SDS–PAGE. The separated proteins on the gels were then transferred to a methanol activated PVDF membrane (0.45 µm; Merck, Darmstadt, Germany) using a Mini Trans-Blot^®^ Cell system (Bio-Rad, Hercules, CA, USA). The membranes were blocked by incubating in 5% milk buffer and then incubated in the following primary target-specific antibodies diluted in 3% bovine serum albumin (BSA, Biomol, Hamburg, Germany) at 4 °C overnight: mouse anti-human HIF-1α (1:250, BD Biosciences, San Jose, CA, USA), mouse anti-VEGF (1:500, Santa Cruz Biotechnology, Dallas, TX, USA), and mouse anti-β-actin (1:10,000, Sigma–Aldrich, Taufkirchen, Germany). On the next day and after washing, the membranes were probed with anti-mouse secondary antibody (1:5000, Dianova, Hamburg, Germany) for 1 h at room temperature. The membranes were developed using the Western Lightning plus-ECL kit (PerkinElmer, Waltham, MA, USA) according to the manufacturer’s protocols.

### 2.5. Bio-Plex Multiplex Immunoassays

After HCAECs reached 70–80% confluency in 6-well plates, they were treated as described for the immunoblotting experiments. The expression of HIF-1α-related proteins in the cell culture medium was analyzed using the Bio-Plex Pro™ Human Cytokine 27-plex kit and a Bio-Plex 200 system according to the manufacturer’s protocols (Bio-Rad, Hercules, CA, USA).

### 2.6. Endothelial Barrier Function Assay

For the trans-endothelial electrical resistance (TEER) measurement and fluorescein isothiocyanate-dextran (FITC-dextran) permeability assay, HCAECs were seeded onto the cell culture insert (transparent membrane, porosity 1 µm, 6-well plates, Greiner Bio-One, Frickenhausen, Germany) at 10 × 10^4^ cells/insert. After three days, HCAECs were treated as described for the immunoblotting experiments. As a positive control for EBD via HIF-1α activity, HCAECs were incubated for 12 and 18 h under hypoxic conditions (2% O_2_ in an incubator chamber, BioSpherix, Parish, NY, USA). Immediately after the treatments, TEER values were measured using an EVOM2 V/ohm meter (World Precision Instruments, Sarasota, FL, USA). In the FITC-dextran permeability assay, after the treatments, HCAECs were cultured with serum-free Endopan 3 medium containing 1 µg/mL FITC-dextran 4-kDa (FD-4, Sigma–Aldrich, St. Louis, MI, USA) for 3 h. The fluorescence of FITC, which had leaked into the lower compartment medium, was then measured using a Synergy HT microplate reader (Bio-Tek Instruments, Winooski, VT, USA).

### 2.7. Immunofluorescence Staining

A total of 12.5 × 10^4^ HCAECs were seeded into the imaging dish (µ-Dish 35 mm, high, ibidi, Gräfelfing, Germany). The treatments were carried out after three days of incubation. The cells were then fixed with freshly prepared 3% paraformaldehyde (PFA) for 10 min at room temperature. After washing, the cells were incubated with phosphate-buffered saline (PBS) containing 10% BSA for 30 min at room temperature. Subsequently, the cells were incubated with mouse anti-human HIF-1α (1:100, BD Biosciences, San Jose, CA, USA), rabbit anti-VE-cadherin (1:200), or rabbit anti-ZO-1 (1:400, Cell Signaling Technology, Leiden, The Netherlands) primary antibodies diluted in PBS with 10% BSA at 4 °C overnight. After washing, the cells were incubated with the secondary antibodies goat anti-mouse Alexa Fluor 594 (1:200, Invitrogen, Thermo Fisher Scientific, Waltham, MA, USA), goat anti-rabbit Alexa Fluor 488 (1:200, Invitrogen, Thermo Fisher Scientific, Waltham, MA, USA), and goat anti-rabbit Alexa Fluor 594 (1:200, Invitrogen, Thermo Fisher Scientific, Waltham, MA, USA) for 1 h at room temperature. During the final washing step, the cells were stained with Hoechst 33342 DNA stain to locate the nucleus. Subsequently, the cells were mounted with mounting medium (ProTaqs^®^ Mount Flour, quartet, Berlin, Germany). Microscopy was carried out using a Zeiss Axiovert 200 m fluorescence microscope equipped with an AxioCam MRm camera and the appropriate filter sets.

### 2.8. Image Processing

Image processing and analysis were performed using the latest version of ImageJ software and the open-source software CellProfiler as we described previously [12,13].

### 2.9. Statistical Analysis

Data were analyzed and represented using GraphPad Prism 9 software (GraphPad Software, San Diego, CA, USA). Experiments were repeated two to four times independently and with different passages of the cells. For sample sizes ≥ 3, the normality and homogeneity of variance were assessed using the Shapiro–Wilk test and an *F* test. The Mann–Whitney U test was used when data did not pass the normality or equal variance tests. For data with a normal distribution, the significant difference between the control group (medium) and treated groups was determined using an unpaired two-tailed Student’s *t* test. For N = 2, an unpaired two-tailed Student’s t test was performed without assessment of the Shapiro–Wilk test or *F* test. Data are given as the mean ± SEM. The results were considered statistically significant if *p* values were less than 0.05 (see Supplementary Table S1 for precise *p* values).

## 3. Results

### 3.1. MFs Induce Normoxic HIF-1α Stabilization in HIF-1α-mKate2-Expressing Cells

**Figure 1:**
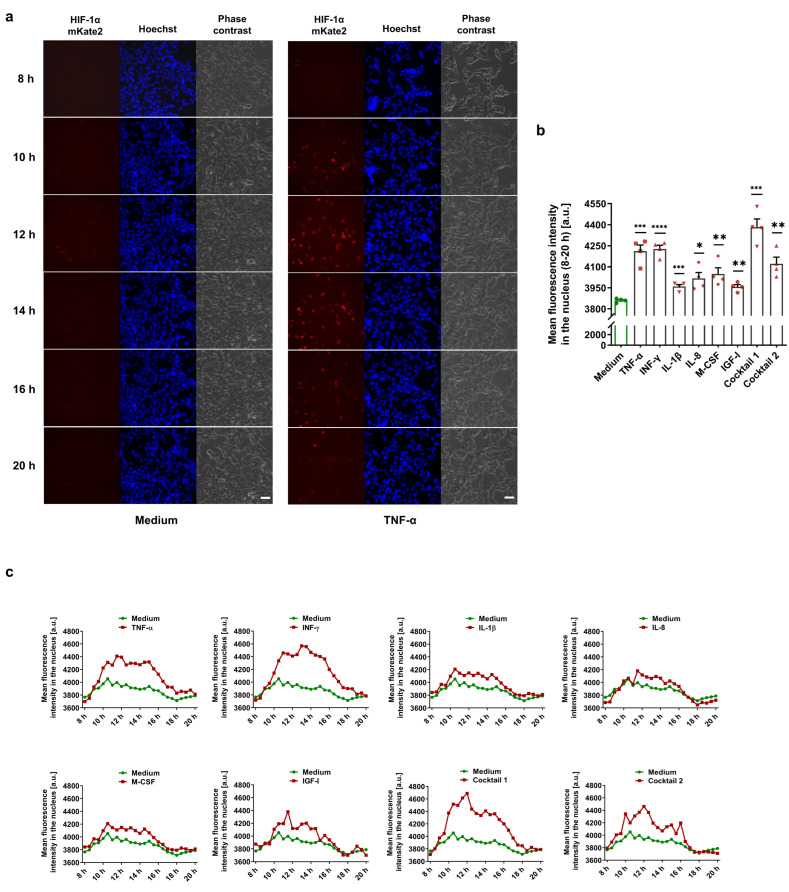
Monitoring of normoxic HIF-1α stabilization in HIF-1α-mKate2-expressing cells. (**a**) Selected frames of live cell imaging upon treatment with 50 ng/mL TNF-α (first column: HIF-1α-mKate2 signal, second column: Hoechst 33342 nuclear staining, and third column: phase contrast; scale bar = 50 µm). TNF-α caused normoxic HIF-1α stabilization in a time-dependent manner (Video S2, see Videos S1–S9 in the Supplementary Material). (**b**) The mean fluorescence intensity deduced from the signal intensities of mKate2 in the nuclei during the time span of 8–20 h of the live cell imaging experiments demonstrates HIF-1α stabilization by the MFs. The data are represented as the mean ± SEM of four independent experiments (N = 4), * *p* value ≤ 0.05, ** *p* value ≤ 0.01, *** *p* value ≤ 0.001, and **** *p* value ≤ 0.0001 vs. medium. (**c**) Time course of HIF-1α stabilization in the treatment groups vs. medium (control group). HIF-1α translocated into the nucleus within 10–20 h after the treatments (see Videos S1–S9 in the Supplementary Material).

To detect HIF-1α-stabilizing MFs in a live cell imaging approach, we used a commercial TALEN donor vector system to generate CMVie-driven HIF-1α-mKate2-expressing HEK-293 cells as a deep red biosensor (Supplementary Figure S1). The knock-in HEK-293 cells were incubated for 24 h in the presence of various MFs under normoxic conditions. HIF-1α stabilization and its translocation into the nucleus were monitored in the control (medium) and treated groups (Videos S1–S9 in the Supplementary Material). We demonstrated that MFs cause normoxic HIF-1α stabilization in a time-dependent manner (Figure 1a). We further analyzed the fluorescence intensity of mKate2 for 8–20 h after the treatments, demonstrating significant HIF-1α stabilization and translocation into the nucleus (Figure 1b,c).

### 3.2. MFs Cause Normoxic HIF-1α Stabilization and Translocation into the Nucleus in HCAECs

**Figure 2:**
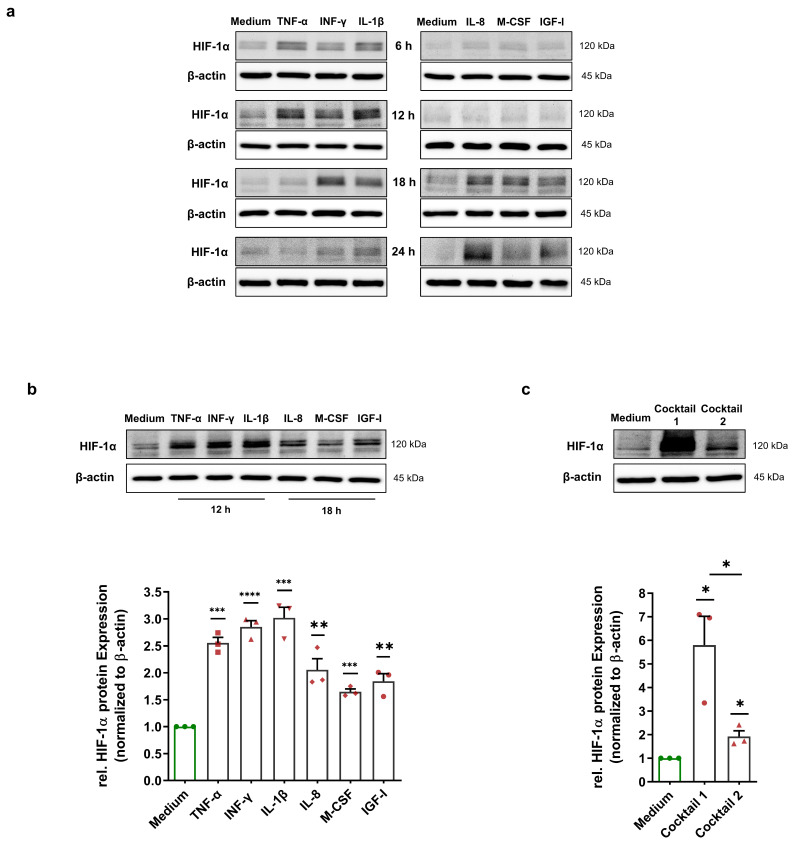
Normoxic HIF-1α stabilization in HCAECs. (**a**) Immunoblots representing HIF-1α protein expression in the total cell lysates of HCAECs incubated with medium (control group) or 50 ng/mL each of TNF-α, INF-γ, IL-1β, IL-8, M-CSF, and IGF-I for 6 h, 12 h, 18 h, and 24 h. The immunoblots show two different time courses of HIF-1α stabilization under normoxic conditions. (**b**) Immunoblots and graphical representation of the fold change of the HIF-1α protein level in the total cell lysates of HCAECs incubated with cell culture medium (control group), 50 ng/mL each of TNF-α, INF-γ, and IL-1β for 12 h or 50 ng/mL each of IL-8, M-CSF, and IGF-I for 18 h. (**c**) Immunoblots and fold change of the HIF-1α protein level upon treatment with 50 ng/mL of cocktail 1 (TNF-α, INF-γ, and IL-1β) for 12 h or 50 ng/mL of cocktail 2 (IL-8, M-CSF, and IGF-I) for 18 h. (**b**,**c**) The data show a significant HIF-1α stabilization upon all treatments. The HIF-1α protein level was normalized to the expression levels of β-actin. The data are represented as the mean ± SEM of three independent experiments (N = 3), * *p* value ≤ 0.05, ** *p* value ≤ 0.01, *** *p* value ≤ 0.001, and **** *p* value ≤ 0.0001 vs. medium.

Immunoblotting was performed to investigate whether MFs could also induce a HIF-1α stabilization in HCAECs. To define the best treatment duration to carry out immunoblotting, HCAECs were cultured with 50 ng/mL preselected HIF-1α-stabilizing MFs (TNF-α, INF-γ, IL-1β, IL-8, M-CSF, and IGF-I) for 6 h, 12 h, 18 h, and 24 h. The level of HIF-1α protein was probed in total cell lysates of treated HCAECs. We demonstrate that the HIF-1α protein increased, peaking within 12 h upon treatment with TNF-α, INF-γ, and IL-1β. Interestingly, treatment with IL-8, M-CSF, and IGF-I showed a different kinetic. The HIF-1α protein level was elevated after 18 h of treatment (Figure 2a). For the 12 and 18 h treatments, we repeated the experiments three times, and immunoblotting was carried out using total cell lysates. Immunoblotting showed a significant upregulation in the HIF-1α protein in all treatment groups (Figure 2b). To determine whether HIF-1α-stabilizing MFs have an additive effect on HIF-1α stabilization, we incubated HCAECs with cocktail 1 containing 50 ng/mL each of TNF-α, INF-γ, and IL-1β for 12 h as well as with cocktail 2 containing IL-8, M-CSF, and IGF-I for 18 h. Interestingly, we observed a strong stabilization (5.79-fold) of the HIF-1α protein in the simultaneous presence of TNF-α, INF-γ, and IL-1β (Figure 2c). In comparison, HIF-1α was stabilized in a nonadditive manner (1.92-fold) upon treatment with the cocktail containing IL-8, M-CSF, and IGF-I (Figure 2c).

**Figure 3:**
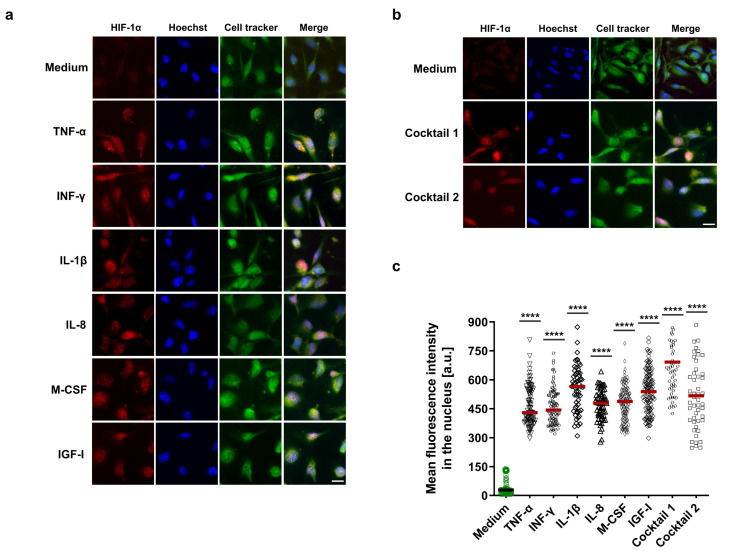
Visualization of HIF-1α localization in the nucleus. (**a**,**b**) Immunofluorescence imaging of HCAECs with antibody against HIF-1α protein (red) in combination with Hoechst 33342 nuclear staining in blue and cell tracker green. Before staining, HCAECs were incubated with 50 ng/mL each of TNF-α, INF-γ, IL-1β, and cocktail 1 (TNF-α, INF-γ, and IL-1β) for 12 h or 50 ng/mL each of IL-8, M-CSF, IGF-I, and cocktail 2 (IL-8, M-CSF, and IGF-I) for 18 h (scale bar = 20 µm). (**c**) The nuclear mean fluorescence intensity in the red channel was measured in the ≤ 60 independent nuclei in the untreated and treated groups. The mean fluorescence intensity increased significantly in the treated groups compared to the control group (medium), **** *p* value ≤ 0.0001 vs. medium.

In addition, immunofluorescence staining was performed to confirm the immunoblotting results as well as visualize HIF-1α localization into the nucleus. HCAECs were treated with HIF-1α-stabilizing MFs, cocktail 1, and cocktail 2. PFA-fixed HCAECs were stained for HIF-1α protein and subsequently analyzed by immunofluorescence microscopy. Consistent with the immunoblotting results, our immunofluorescence data display highly significant HIF-1α stabilization and its localization in the nucleus in all treatment groups as well as an additive effect for cocktail 1 (Figure 3a–c).

### 3.3. Normoxic HIF-1α Stabilization Leads to the Upregulation of HIF-1α-Related Proteins

**Figure 4:**
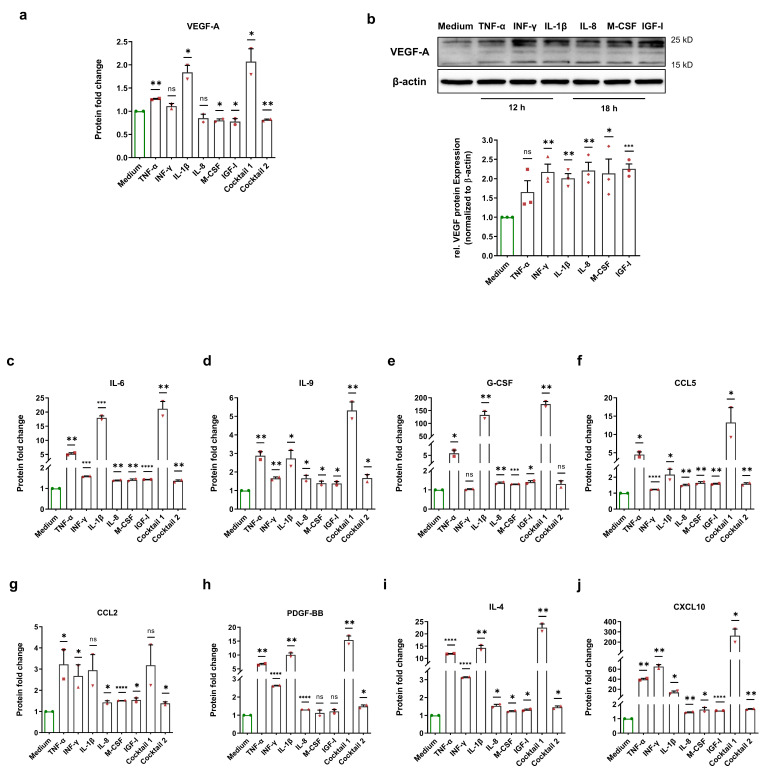
Enhancement of HIF-1α-related proteins after normoxic HIF-1α stabilization. (**a**) Fold change of the VEGF-A protein level in the cell culture medium of HCAECs incubated with cell culture medium (control group), 50 ng/mL each of TNF-α, INF-γ, IL-1β, and cocktail 1 (TNF-α, INF-γ, and IL-1β) for 12 h or 50 ng/mL each of IL-8, M-CSF, IGF-I, and cocktail 2 (IL-8, M-CSF, and IGF-I) for 18. Normoxic HIF-1α stabilization only led to an increase in the VEGF-A protein level in the cell culture medium of HCAECs treated with TNF-α, INF-γ, IL-1β, and cocktail 1 (TNF-α, INF-γ, and IL-1β). The data are represented as the mean ± SEM of two independent experiments (N = 2), ns = not significant, * *p* value ≤ 0.05 and ** *p* value ≤ 0.01 vs. medium. (**b**) Immunoblots and graphical representation of the fold change of the VEGF-A protein level in the total cell lysates of HCAECs incubated with cell culture medium (control group), 50 ng/mL each of TNF-α, INF-γ, and IL-1β for 12 h or 50 ng/mL each of IL-8, M-CSF, and IGF-I for 18 h. The data show an increase in the VEGF-A protein level inside of HCAECs upon all treatments. The data are represented as the mean ± SEM of three independent experiments (N = 3), ns = not significant, * *p* value ≤ 0.05, ** *p* value ≤ 0.01, and*** *p* value ≤ 0.001 vs. medium. (**c–j**) After the incubation of HCAECs with the medium (control group), 50 ng/mL each of TNF-α, INF-γ, IL-1β, and cocktail 1 (TNF-α, INF-γ, and IL-1β) for 12 h or 50 ng/mL each of IL-8, M-CSF, IGF-I, and cocktail 2 (IL-8, M-CSF, and IGF-I) for 18, the cell culture medium of HCAECs was analyzed to measure the protein fold change of (**c**) IL-6, (**d**) IL-9, (**e**) G-CSF, (**f**) CCL5, (**g**) CCL2, (**h**) PDGF-BB, (**i**) IL-4, and (**j**) CXCL10. The data are represented as the mean ± SEM of two independent experiments (N = 2), ns = not significant, * *p* value ≤ 0.05, ** *p* value ≤ 0.01, *** *p* value ≤ 0.001, and **** *p* value ≤ 0.0001 vs. medium.

To examine the consequences of normoxic HIF-1α stabilization, we incubated HCAECs with HIF-1α-stabilizing MFs, cocktail 1 (TNF-α, INF-γ, and IL-1β), and cocktail 2 (IL-8, M-CSF, and IGF-I) for 12 h and 18 h. Next, Bio-Plex assays from the cell culture medium of each treatment were performed to evaluate various HCAECs-released HIF-1α-related proteins. Vascular endothelial growth factor (VEGF)-A is one of the key target genes of HIF-1α, and plays a crucial role in angiogenesis as well as in the development of atherosclerosis [14,15]. We observed an upregulation of the VEGF-A protein level in the cell culture medium of treated HCAECs with TNF-α, INF-γ, IL-1β, and cocktail 1 (TNF-α, INF-γ, and IL-1β). However, we did not observe this alteration in the cell culture medium upon other treatments (Figure 4a). To test whether the VEGF-A protein is upregulated inside of HCAECs, we carried out immunoblotting using the total cell lysates of HCAECs incubated with HIF-1α-stabilizing MFs. Immunoblotting revealed an increase in the VEGF-A protein level upon all treatments (Figure 4b).

In addition, it has been reported that the increase in HIF-1α activity induces an upregulation of IL-6 protein. Conversely, inhibition of HIF-1α using 2-methoxyestradiol significantly decreased IL-6 expression [16]. Our data indicates a significant enhancement of IL-6 in the cell culture medium (Figure 4c). In a recent study, Roy et al. showed that HIF-1α is involved in IL-9 production [17]. In line with their findings, we demonstrate that the IL-9 protein level increased after normoxic HIF-1α stabilization (Figure 4d). Moreover, HIF-1α induces carbonic anhydrase IX (CAIX) expression and thereby increases G-CSF expression via the CAIX/NF-κB/G-CSF cellular signaling axis [18]. Analysis of the HCAECs-derived secretome revealed an increase in G-CSF protein after normoxic HIF-1α stabilization (Figure 4e).

Furthermore, HIF-1α is involved in the hypoxia-stimulated expression of CC chemokines such as CCL2 or monocyte chemotactic protein 1 (MCP-1) and CCL5 or RANTES [19,20]. Under normoxia, our data show a significant upregulation of the CCL5 protein in all treatment groups as well as a significant upregulation of the CCL2 protein upon most treatments (Figure 4f,g). Semenza et al. discovered that *PDGF-B* is a direct HIF-1α target gene and that hypoxia increases the PDGF-BB protein level [21]. Here, we show that the PDGF-BB protein level increased after normoxic HIF-1α stabilization (Figure 4h). In addition, Yang et al. reported the involvement of HIF-1α in IL-4 induction [22]. Our data show that IL-4 was significantly increased in the cell culture medium upon all treatments (Figure 4i). C-X-C motif ligand 10 (CXCL10) or interferon-inducible protein-10 (IP-10) is a small chemokine with potent chemotactic activity on immune cells and is upregulated under hypoxic conditions [23]. We found a significant enhancement of the CXCL10 protein in all treatment groups (Figure 4j). In line with the current literature, we demonstrate an induction of HIF-1α-dependent soluble factors, and the expression level correlates with our immunoblotting findings for the two MFs groups.

### 3.4. Normoxic HIF-1α Stabilization and Upregulation of HIF-1α-Related Proteins Could Promote Endothelial Barrier Dysfunction

**Figure 5:**
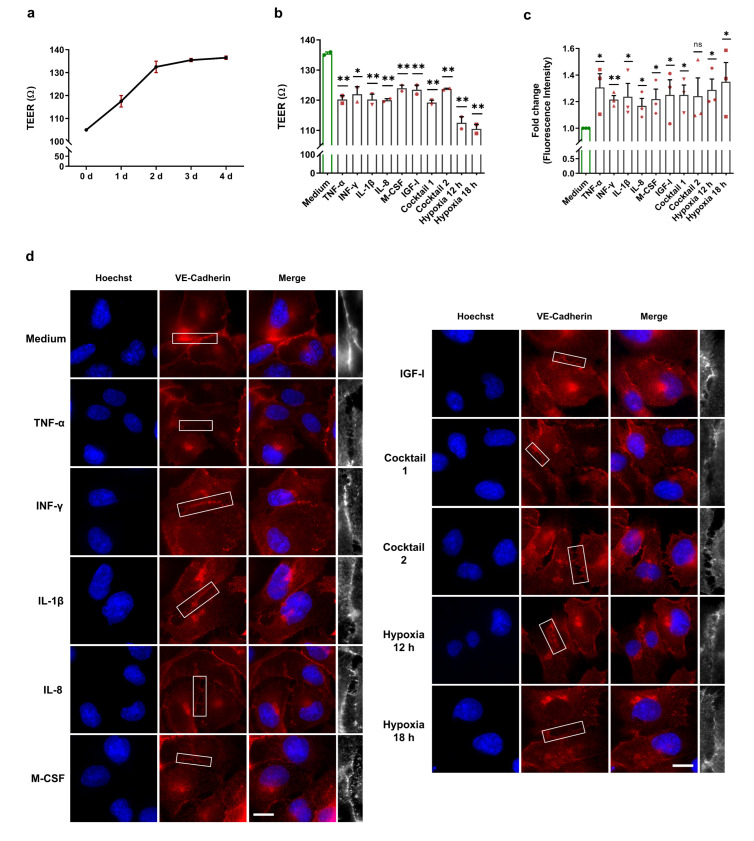

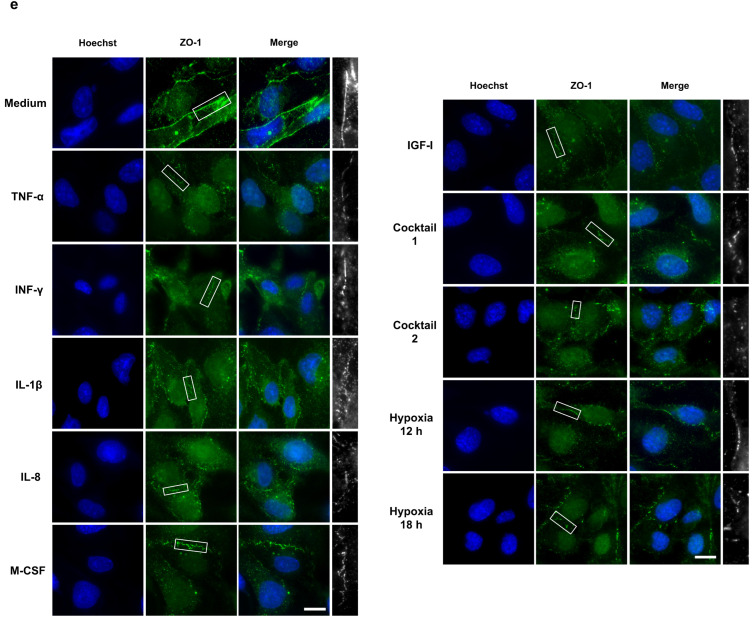
Altered endothelial barrier integrity of HCAECs after HIF-1α stabilization under normoxic conditions. (**a**) Daily TEER measurements were conducted after cell seeding and reached a stable TEER value of approximately 135 Ω. The graphic represents two independent experiments (N = 2). (**b**–**e**) For the endothelial barrier integrity experiments, HCAECs were incubated with cell culture medium (control group), 50 ng/mL each of TNF-α, INF-γ, IL-1β, and cocktail 1 (TNF-α, INF-γ, and IL-1β) for 12 h or 50 ng/mL each of IL-8, M-CSF, IGF-I, and cocktail 2 (IL-8, M-CSF, and IGF-I) for 18 h. As a positive control for EBD caused by HIF-1α activation, the HCAECs monolayer was incubated for 12 h and 18 h under hypoxic conditions. (**b**) TEER measurement of the HCAECs monolayer and graphical representation of changes in the TEER values in the untreated and treated groups. The data show significant EBD with all treatments and under hypoxic conditions. The data are represented as the mean ± SEM of two independent experiments (N = 2), * *p* value ≤ 0.05 and ** *p* value ≤ 0.01 vs. medium. (**c**) Leaked FITC-dextran was measured in the untreated and treated groups. The graphic represents the fold change in the measured fluorescence intensity in the acceptor (cell culture medium). These data confirm the barrier dysfunction upon all treatments. The data are represented as the mean ± SEM of three independent experiments (N = 3), ns = not significant, * *p* value ≤ 0.05 and ** *p* value ≤ 0.01 vs. medium. (**d**,**e**) Immunofluorescence imaging of HCAECs with antibodies against VE-cadherin (red) (**d**) and against ZO-1 (green) (**e**) in combination with Hoechst 33342 nuclear staining (blue). ZO-1 and VE-cadherin staining depicts the EBD in all treatment groups, as well as under hypoxia (scale bar = 20 µm). The last column (white) is the magnification of the selected barrier to illustrate clearly EBD.

EBD is the first trigger for the development of atherosclerosis through uncontrolled leukocyte extravasation into the intima. Previous studies discovered the crucial role of HIF-1α activation in EBD [24,25,26]. To determine endothelial barrier integrity upon our treatments and after normoxic HIF-1α stabilization, we performed TEER measurements and FITC-dextran permeability assays as well as immunofluorescence staining, which are three widely accepted techniques for EBD analysis. To check HCAECs monolayer formation, we carried out daily TEER measurements after the cells were seeded into the transwell cell inserts. As illustrated in Figure 5a, after three to four days, HCAECs formed a monolayer and reached a stable TEER value of approximately 135 Ω. For our endothelial barrier integrity experiments, we cultured HCAECs for three days and then treated them with 50 ng/mL of each HIF-1α-stabilizing MFs, cocktail 1 (TNF-α, INF-γ, and IL-1β), and cocktail 2 (IL-8, M-CSF, and IGF-I) for 12 h and 18 h. As a positive control for EBD caused by HIF-1α activation, we incubated HCAECs monolayers for 12 h and 18 h under hypoxic conditions. We demonstrate significant EBD in the HCAECs stimulated with the HIF-1α-stabilizing MFs and the cocktails (Figure 5b). However, hypoxia caused more severe endothelial dysfunction (Figure 5b). Next, we determined the EBD in HCAECs by using FITC-dextran assays. The FITC-dextran data confirm the barrier dysfunction upon our treatments as well as significant EBD under hypoxia (Figure 5c). To visualize the proven barrier dysfunction, PFA-fixed HCAECs were stained for cell junction proteins (ZO-1 and VE-cadherin) and subsequently analyzed by immunofluorescence microscopy. In line with the results from the TEER and FITC-dextran experiments, ZO-1 and VE-cadherin staining visualized EBD in all treatment groups, as well as under hypoxia (Figure 5d,e).

## 4. Discussion

The mechanisms and circumstances that lead to HIF-1α stabilization under normoxic conditions are still uncharted. Therefore, we aimed to reveal the HIF-1α-stabilizing capabilities of various MFs and analyze their effect on endothelial barrier function. HIF-1α stabilization is a time-dependent process. To facilitate kinetic studies of normoxic HIF-1α activation and to be able to distinguish between internal and external (HIF-1α-mKate2) HIF-1α protein, we generated HIF-1α-mKate2-expressing HEK-293 cells using the TALEN genome editing system. In this work, we demonstrate that proinflammatory cytokines such as TNF-α caused significant normoxic HIF-1α stabilization in HCAECs, which is in line with previous studies showing normoxic HIF-1α stabilization caused by TNF-α in HEK-293 cells and airway smooth muscle cells [27,28]. Under normoxia, INF-γ was able to stabilize HIF-1α in various cancer cell lines and in human aortic valve interstitial cells [9,29]. We also found that INF-γ induces normoxic HIF-1α stabilization in HIF-1α-mKate2-expressing HEK-293 cells and HCAECs. In addition, induction of HIF-1α protein by IL-1β and IGF-I has been reported in other cell lines, such as human osteoarthritic chondrocytes, cytotrophoblast cells, human gingival and synovial fibroblasts, and colon and breast cancer cells [30,31,32,33,34]. In line with the abovementioned previous studies, our data demonstrate elevated HIF-1α protein in the presence of IL-1β and IGF-I in both HIF-1α-mKate2-expressing HEK-293 cells and HCAECs. To the best of our knowledge, the present study shows for the first time that IL-8 and M-CSF induce HIF-1α stabilization under normoxic conditions.

Furthermore, our results revealed for the first time two different kinetics of HIF-1α protein level increase under normoxic conditions. Whereas treatments with TNF-α, INF-γ, and IL-1β caused normoxic HIF-1α stabilization stronger and already after 6 h (peaking within 12 h), treatments with IL-8, M-CSF, and IGF-I stabilized HIF-1α more weakly, and with a peak after 18 h. During a wide range of diseases such as acute respiratory distress syndrome (ARDS), COVID-19 infection, or in sepsis the level of circulating cytokines are increased [35,36]. Therefore, in order to determine whether the simultaneous presence of HIF-1α-stabilizing MFs have an additive effect on HIF-1α stabilization, we used two cocktails in this work. Interestingly and for the first time, we demonstrate that treatment with cocktail 1 consisting of TNF-α, INF-γ, and IL-1β resulted in a cumulative increase in HIF-1α protein (5.79-fold) compared to a single treatment with TNF-α (2.55-fold), INF-γ (2.84-fold), and IL-1β (3.03-fold). Conversely, the simultaneous presence of IL-8, M-CSF, and IGF-I (cocktail 2) did not result in an additive increase in HIF-1α protein compared to single treatments with each of these MFs. This finding, together with our results on different kinetics of HIF-1α stabilization upon our treatments, suggest that there are at least two different patterns or mechanisms to stabilize HIF-1α protein under normoxic conditions.

Endothelial HIF-1α has been shown to promote atherosclerosis not only through its role in barrier dysfunction, but also by modulating other processes of progression of atherosclerosis such as monocyte recruitment [4]. For this reason, and in contrast to previous in vitro studies, we analyzed the protein levels of HIF-1α-related factors in the environment (cell culture medium) of HCAECs. VEGF-A is one of the most important HIF-1α-dependent target genes in cardiovascular disease. In our previous report, we showed that oxidized-LDL enhances normoxic HIF-1α DNA-binding activity, which upregulates the mRNA expression of VEGF-A [8]. Here, we identified enhancement of VEGF-A protein levels both within the cell culture medium and in HCAECs upon treatment with TNF-α, INF-γ, and IL-1β. However, treatment with IL-8, M-CSF, and IGF-I only increased VEGF-A protein levels intracellularly. This could be explained by the different time courses of the transcription, translation, and release of these soluble factors. To be in line with the rest of our data, we chose the time points for analysis based on the HIF-1α stabilization peaks, and they were not optimized for the expression of the HIF-1α target proteins.

Earlier studies revealed that endothelial VEGF induces dysfunction and migration of ECs and also increases migration and proliferation of VSMCs [37,38,39]. Our data demonstrate EBD upon our treatments and under hypoxia. In addition to MFs, elevated VEGF-A protein is a crucial factor involved in EBD and acts as one of the earliest triggers of atherosclerosis [15,25,37]. The secreted endothelial VEGF-A might also play an important role in the progression of atherosclerosis by promoting VSMCs migration.

To further demonstrate HIF-1α dependent transactivation, we analyzed the HCAECs-derived secretome. In particular, we investigated the expression and secretion of known HIF-1α-related proteins under normoxic condition and after HIF-1α stabilization. Numerous studies have reported the involvement of HIF-1α in the production of IL-4, IL-6, IL-9, G-CSF, CCL2, CCL5, CXCL10, and PDGF-BB [17,18,19,20,21,22,23]. Although IL-4 was traditionally classified as an anti-inflammatory cytokine, previous works discovered that IL-4 is involved in the development of atherosclerosis, with a proinflammatory role in EBD and enhancement of monocyte recruitment through the upregulation of vascular cell adhesion molecule-1 (VCAM-1) in ECs [40,41]. Here, we identified a significant increase in IL-4 protein upon our treatments and after normoxic HIF-1α stabilization. Together with VEGF-A, IL-4 may play an important role in endothelial dysfunction in HCAECs. The increased IL-4 level might also aggravate the progression of atherosclerosis through its role in monocyte recruitment via the upregulation of VCAM-1 in ECs.

Moreover, we observed enhancement of the IL-6 protein in the HCAECs’ environment. In addition to MFs, IL-6 could be another cause of EBD, as shown in early studies [42,43]. IL-6 also promotes the upregulation of HIF-1α expression and enhances the translocation of HIF-1α into the nucleus [44,45]. It might form a HIF-1α/IL-6/HIF-1α positive feedback loop, resulting in a vicious cycle promoting the progression of atherosclerosis via endothelial HIF-1α activation. Our secretome analysis also showed an increase in the G-CSF protein. Since G-CSF can cause normoxic HIF-1α stabilization [46], a feedback loop between G-CSF and HIF-1α might also be formed, which could further accelerate the progression of atherosclerosis.

Furthermore, regulation of IL-9 via HIF-1α was described in a recent work [17]. In line with this work, we significantly demonstrated the upregulation of IL-9 upon our treatments and after normoxic HIF-1α stabilization. Endothelial IL-9 accelerates the formation of atherosclerotic plaques via upregulation of VCAM-1 as well as via enhancement of monocyte recruitment, as revealed by Zhang et al. in ApoE^−/−^ mice [47].

Chemokines such as CCL2, CCL5, and CXCL10 play a crucial role in the early stages of atherosclerosis via EBD as well as via atherogenic phenotype switching of VSMCs [48,49,50]. Analysis of the endothelial secretome and immunofluorescence staining revealed an increase in CCL2 after normoxic HIF-1α stabilization as well as ZO-1 dysfunction. These data are in line with a previous study by Stamatovic et al., who reported ZO-1 dysfunction via CCL2 [49]. CXCL10 and CLL5 protein levels were also increased in the HCAECs-derived secretome, which could aggravate the progression of atherosclerosis by promoting the proliferation and atherogenic phenotype switching of VSMCs.

Certainly, the mentioned mediators are not only upregulated via HIF-1α activity. However, in addition to other possible pathways that can be activated by MFs, numerous studies have reported the crucial role of HIF-1α stabilization in the regulation of these proinflammatory mediators. In line with the current literature, our secretome analysis showed an induction of HIF-1α-dependent soluble factors in the presence of HIF-1α stabilizing MFs and their expression level correlated with our immunoblotting findings for the HIF-1α stabilization upon treatments with two MFs groups. These mediators could aggravate the progression of atherosclerosis through promotion of EBD, VSMCs migration/proliferation, and through their role in monocyte recruitment, as mentioned above for each secreted mediator.

**Figure 6:**
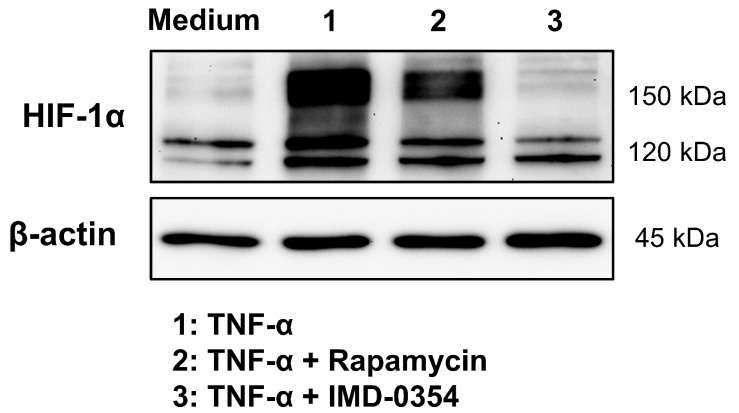
NF-κB regulates HIF-1α stabilization. Immunoblot representing HIF-1α protein expression in the total cell lysates of HIF-1α-mKate2-expressing HEK-293 cells incubated with cell culture medium (control group), 50 ng/mL of TNF-α, 50 ng/mL of TNF-α and 150 nmol/L of rapamycin, or 50 ng/mL of TNF-α and 1 µmol/L of IMD-0354 for 12 h. Inhibition of NF-κB signaling pathway led to downregulation of internal HIF-1α protein (about 120 kDa). The treatment of cells with TNF-α and rapamycin caused a weak decrease in HIF-1α-mKate2 protein level, whose gene is located on chromosome 19 and CMVie-driven (about 160 kDa). Inhibition of the NF-κB signaling pathway using IMD-0354 led to complete degradation of HIF-1α-mKate2 protein (about 160 kDa). The data display an indirect regulation of HIF-1α protein stability by NF-κB activity.

In the current literature, the activation of the NF-κB, mTOR, and STAT1/3 pathways is reported as possible signaling pathways for HIF-1α stabilization under normoxic conditions. Above all, a direct upregulation of HIF-1α mRNA and protein has been demonstrated through NF-κB stabilization and activity in previous works [9,27,29,44,51]. In contrast to the HIF-1α transcript upregulation, our CMVie promotor-driven HIF-1α-mKate2 reporter, demonstrates a stabilization, which is independent from a direct NF-κB transactivation, since both the native HIF-1α promotor and the 5′ and 3′ untranslated regulatory regions of the mRNA are missing in the knock-in situation. Therefore, stabilized HIF-1α-mKate2 protein cannot be the consequence of a direct RNA upregulation by NF-κB. IMD-0354 is a selective IKK-β inhibitor, which inhibits NF-κB activity and is currently in phase 2 clinical trial [52]. Treatment of HIF-1α-mKate2-expressing knock-in cells with TNF-α and IMD-0354 showed not only a downregulation of HIF-1α protein level, but also a very strong decrease in HIF-1a-mKate2 protein (Figure 6). This finding, together with our results from live cell imaging experiments, indicates that, in addition to the known direct regulation of HIF-1α by NF-κB, there is a hitherto unknown indirect machinery which is regulated by NF-κB causing the strong increase in HIF-1α-mKate2 protein. Future work will address this indirect mechanism. The signaling pathways that stabilize HIF-1α under normoxic conditions should also be further studied by characterizing the two different kinetics we described. In particular, the signaling pathways of hitherto unknown factors of normoxic HIF-1α stabilization (IL-8 and M-CSF) as well as the cause of the accumulative effect on HIF-1α stabilization upon treatment with cocktail 1 (TNF-α, INF-γ, and IL-1β) will be investigated. Moreover, to clarify the precise role of normoxic HIF-1α stabilization and MFs in EBD, barrier integrity of HCAECs should be further investigated in the presence of inhibitors of NF-κB, mTOR, and STAT1/3 signaling pathways, as well as in presence of anti HIF-1α siRNA or in HIF-1α-knock-out cells.

Furthermore, we only used a monoculture of HCAECs. Under physiological conditions, HCAECs interact with many other cells, such as VSMCs, local and circulating immune cells, and the extracellular matrix. Therefore, our results need to be investigated in more complex systems, such as a microfluidic organoid-on-a-chip model, as well as in vivo models, such as ApoE^−/−^ mice. The results of these studies reveal new insights into the role of normoxic endothelial HIF-1α stabilization and its putative role in the development and progression of atherosclerosis, which could offer novel therapeutic options for plaque prevention/regression using modulators of HIF-1α-mediated gene transcription.

## Data Availability

Raw data were generated at Otto-von-Guericke University. Derived data supporting the findings of this study are available from the corresponding author R.C.B.-D. and S.W. upon reasonable request.

## Supplementary Materials

The following supporting information can be downloaded at: https://www.mdpi.com/article/10.3390/cells11233878/s1, Figure S1: Generation of the HIF-1α biosensor; Figure S2: Illustration of a selected frame of live cell imaging video; Table S1: Details of the statistical tests performed; Live cell imaging Videos S1–S9; original immunoblots.

## Abbreviations

EBD	Endothelial barrier dysfunction
ECs	Endothelial cells
FITC-dextran	Fluorescein isothiocyanate-dextran
HCAECs	Human coronary endothelial cells
HEK-293 cells	Human embryonic kidney 293 cells
IGF-I	Insulin-like growth factor-I
IL	Interleukin
INF-γ	Interferon-γ
M-CSF	Macrophage colony-stimulating factor
MFs	Micromilieu factors
TEER	Transendothelial electrical resistance
TNF-α	Tumor necrosis factor-α
VSMCs	Vascular smooth muscle cells
