# Supplementary material for: Normoxic HIF-1α Stabilization Caused by Local Inflammatory Factors and Its Consequences in Human Coronary Artery Endothelial Cells
Source: Cells. 2022 Dec 1;11(23):3878. doi: 10.3390/cells11233878 (PMC9737288; doi:10.3390/cells11233878)

Supplemental Material

Normoxic HIF-1α Stabilization Caused by Local Inflammatory Factors and Its Consequences in Human Coronary Artery Endothelial Cells

Mohsen Abdi Sarabi [1], Alireza Shiri [1], Mahyar Aghapour [1,2,†], Charlotte Reichardt [3], Sabine Brandt [3], Peter R. Mertens [3], Senad Medunjanin [1], Dunja Bruder [2,4], Ruediger C. Braun-Dullaeus [1,*,‡] and Sönke Weinert [1,*,‡]

[1] Department of Internal Medicine, Division of Cardiology and Angiology, Otto-von-Guericke University, 39120 Magdeburg, Germany
[2] Infection Immunology Group, Institute of Medical Microbiology and Hospital Hygiene, Otto-von-Guericke University, 39120 Magdeburg, Germany
[3] Clinic of Nephrology and Hypertension, Diabetes and Endocrinology, Otto-von-Guericke University, 39120 Magdeburg, Germany
[4] Immune Regulation Group, Helmholtz Centre for Infection Research, 38124 Braunschweig, Germany
***** Correspondence: r.braun-dullaeus@med.ovgu.de (R.C.B.-D.); soenke.weinert@med.ovgu.de (S.W.)
† Current Adress: Department of Dermatology and Allergic Diseases, Ulm University, 89081 Ulm, Germany.
‡ These authors contributed equally to this work.

1. Supplementary methods

1.1 Preparation of the donor vector

HIF-1α cds excluding the stop codon was fused to mKate2 using pmKate2-N (Evrogen, Moscow, Russia) and further subcloned into the commercial AAVS1 TALE-Nuclease Kit donor vector (System Biosciences, Palo Alto, USA). The donor vector was additionally modified by exchange of the PGK promotor for a CMVie promotor and the exchange of puromycin resistance for hygromycin resistance. PCR fragments of the CMVie promotor and hygromycin resistance were created using pShuttle (Takara Bio, Kusatsu Japan) and pGL4.32 (Promega, Madison, USA) as templates and cloned via assembly cloning using an In-Fusion cloning kit (Takara Bio, Kusatsu Japan).

1.2 Confirmation of stable genomic integration into the AAVS1 locus (junction PCR)

After transfection of the vector into HEK293 cells, stable genomic integration into the AAVS1 locus on chromosome 19 was confirmed by junction PCR. For this, genomic DNA (gDNA) was extracted from transfected HEK293 cells. The flanking regions 3' and 5' of the TALE nuclease cut site at the AAVS1 locus were amplified using two primer groups (right and left). Each primer group consisted of a primer for the original genomic sequence and a primer within the donor vector sequence. PCRs were carried out according to the manufacturer's protocols (System Biosciences, Palo Alto, USA). The PCR products could only be expected if the integration was successful (Supplementary Figure 1c).

a

pAAVS1D CMV HIF-1α-mKate2 copGFP T2A HygroR

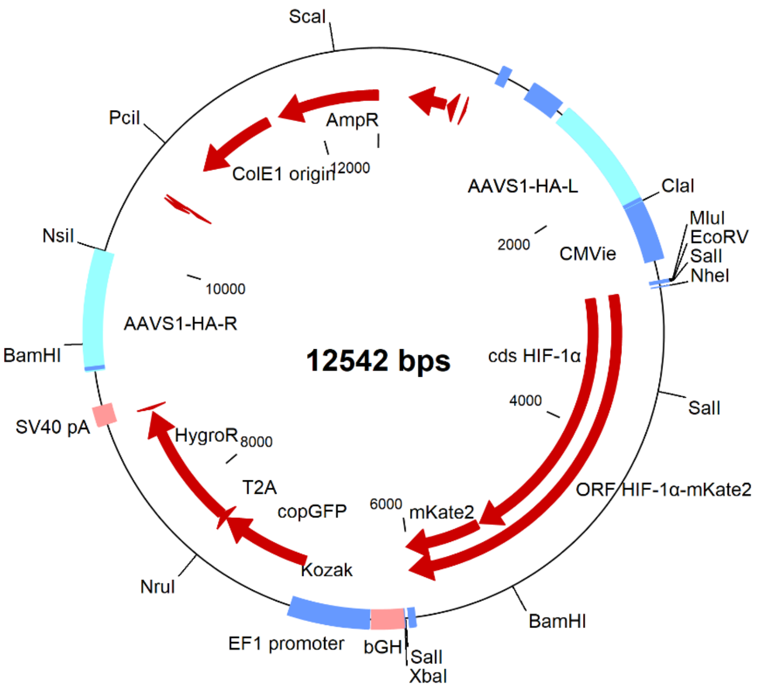

b

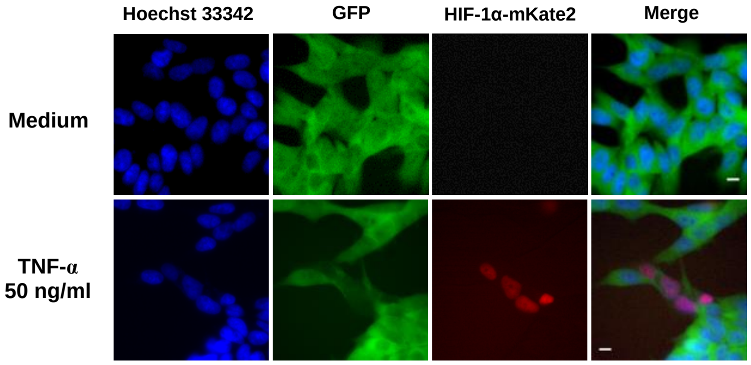

c

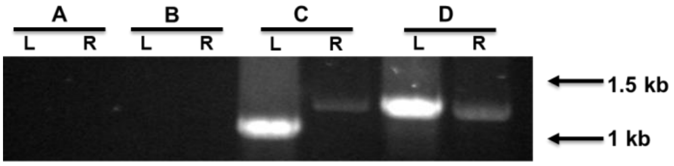

Supplementary Figure S1. Generation of the HIF-1α biosensor. a) Vector map used for the generation of HIF-1α biosensor. **b)** Hoechst 33342 nuclear staining (blue), green fluorescent protein (GFP) produced by cells (green) and stabilized HIF-1α-mKate2 in the nucleus (red). HIF-1α-mKate2 expressing cells were incubated with the cell culture medium and with 50 ng/ml TNF-α for 12 h. TNF-α caused normoxic HIF-1α stabilization (scale bar = 10 µm). **c)** Junction PCR products. A: wt-HEK293 cells (negative control), B: HEK293-cells transfected with pAAVS1D-CMV-RFP-GFP (negative control), C: HEK293 cells transfected with pAAVS1D- CMV-RFP-GFP and pZT-AAVS1-L1/R1 (positive control), D: HEK293 cells transfected with pAAVS1D-HIF-1α-mKate2-GFP and pZT-AAVS1-L1/R1. The PCR products can only be expected if the integration into AAVS1 is successful (C and D).

1.3 Statistical analysis

Supplementary Table S1. Details of the statistical tests performed.

Figure	Type of test	Sample size	*p value*
**1 b**	Unpaired Student's t test	N = 4	Medium vs. TNF-α = **0.0002**<br>Medium vs. INF-γ = **<0.0001**<br>Medium vs. IL-1β = **0.0009**<br>Medium vs. IL-8 = **0.0104**<br>Medium vs. M-CSF = **0.0058**<br>Medium vs. IGF-I = **0.0014**<br>Medium vs. cocktail 1 (TNF-α, INF-γ and IL-1β) = **0.0001**<br>Medium vs. cocktail 2 (IL-8, M-CSF and IGF-I) = **0.0015**
**2 b**	Unpaired Student's t test	N = 3	Medium vs. TNF-α = **0.0001**<br>Medium vs. INF-γ = **<0.0001**<br>Medium vs. IL-1β = **0.0005**<br>Medium vs. IL-8 = **0.0070**<br>Medium vs. M-CSF = **0.0002**<br>Medium vs. IGF-I = **0.0041**
**2 c**	Unpaired Student's t test	N = 3	Medium vs. cocktail 1 (TNF-α, INF-γ and IL-1β) = **0.0173**<br>Medium vs. cocktail 2 (IL-8, M-CSF and IGF-I) = **0.0205**<br>Cocktail 1 (TNF-α, INF-γ and IL-1β) vs. cocktail 2 (IL-8, M-CSF and IGF-I) = **0.0362**
**3 c**	Mann–Whitney test	N ≤ 60	Medium vs. TNF-α = **<0.0001**<br>Medium vs. INF-γ = **<0.0001**<br>Medium vs. IL-1β = **<0.0001**<br>Medium vs. M-CSF = **<0.0001**<br>Medium vs. cocktail 1 (TNF-α, INF-γ and IL-1β) = **<0.0001**<br>Medium vs. cocktail 2 (IL-8, M-CSF and IGF-I) = **<0.0001**
	Unpaired Student's t test	N ≤ 60	Medium vs. IL-8 = **<0.0001**<br>Medium vs. IGF-I = **<0.0001**
**4 a**	Unpaired Student's t test	N = 2	Medium vs. TNF-α = **0.0013**<br>Medium vs. INF-γ = **0.0930**<br>Medium vs. IL-1β = **0.0149**<br>Medium vs. IL-8 = **0.1120**<br>Medium vs. M-CSF = **0.0120**<br>Medium vs. IGF-I = **0.0372**<br>Medium vs. cocktail 1 (TNF-α, INF-γ and IL-1β) = **0.0307**<br>Medium vs. cocktail 2 (IL-8, M-CSF and IGF-I) = **0.0046**

**4 b**	Unpaired Student's t test	N = 3	Medium vs. TNF-α = **0.0873**<br>Medium vs. INF-γ = **0.0042**<br>Medium vs. IL-1β = **0.0012**<br>Medium vs. IL-8 = **0.0046**<br>Medium vs. M-CSF = **0.0360**<br>Medium vs. IGF-I = **0.0005**
**4 c**	Unpaired Student's t test	N = 2	Medium vs. TNF-α = **0.0038**<br>Medium vs. INF-γ = **0.0004**<br>Medium vs. IL-1β = **0.0008**<br>Medium vs. IL-8 = **0.0011**<br>Medium vs. M-CSF = **0.0078**<br>Medium vs. IGF-I = **<0.0001**<br>Medium vs. cocktail 1 (TNF-α, INF-γ and IL-1β) = **0.0079**<br>Medium vs. cocktail 2 (IL-8, M-CSF and IGF-I) = **0.0095**
**4 d**	Unpaired Student's t test	N = 2	Medium vs. TNF-α = **0.0062**<br>Medium vs. INF-γ = **0.0061**<br>Medium vs. IL-1β = **0.0277**<br>Medium vs. IL-8 = **0.0256**<br>Medium vs. M-CSF = **0.0341**<br>Medium vs. IGF-I = **0.0286**<br>Medium vs. cocktail 1 (TNF-α, INF-γ and IL-1β) = **0.0054**<br>Medium vs. cocktail 2 (IL-8, M-CSF and IGF-I) = **0.0372**
**4 e**	Unpaired Student's t test	N = 2	Medium vs. TNF-α = **0.0147**<br>Medium vs. INF-γ = **0.1318**<br>Medium vs. IL-1β = **0.0055**<br>Medium vs. IL-8 = **0.0092**<br>Medium vs. M-CSF = **0.0005**<br>Medium vs. IGF-I = **0.0167**<br>Medium vs. cocktail 1 (TNF-α, INF-γ and IL-1β) = **0.0016**<br>Medium vs. cocktail 2 (IL-8, M-CSF and IGF-I) = **0.1022**
**4 f**	Unpaired Student's t test	N = 2	Medium vs. TNF-α = **0.0189**<br>Medium vs. INF-γ = **<0.0001**<br>Medium vs. IL-1β = **0.0365**<br>Medium vs. IL-8 = **0.0066**<br>Medium vs. M-CSF = **0.0067**<br>Medium vs. IGF-I = **0.0018**<br>Medium vs. cocktail 1 (TNF-α, INF-γ and IL-1β) = **0.0474**<br>Medium vs. cocktail 2 (IL-8, M-CSF and IGF-I) = **0.0069**
**4 g**	Unpaired Student's t test	N = 2	Medium vs. TNF-α = **0.0425**<br>Medium vs. INF-γ = **0.0430**<br>Medium vs. IL-1β = **0.0591**<br>Medium vs. IL-8 = **0.0152**<br>Medium vs. M-CSF = **<0.0001**<br>Medium vs. IGF-I = **0.0148**

			Medium vs. cocktail 1 (TNF-α, INF-γ and IL-1β) = **0.0750**<br>Medium vs. cocktail 2 (IL-8, M-CSF and IGF-I) = **0.0154**
**4 h**	Unpaired Student's t test	N = 2	Medium vs. TNF-α = **0.0016**<br>Medium vs. INF-γ = **<0.0001**<br>Medium vs. IL-1β = **0.0028**<br>Medium vs. IL-8 = **<0.0001**<br>Medium vs. M-CSF = **0.2572**<br>Medium vs. IGF-I = **0.0942**<br>Medium vs. cocktail 1 (TNF-α, INF-γ and IL-1β) = **0.0051**<br>Medium vs. cocktail 2 (IL-8, M-CSF and IGF-I) = **0.0123**
**4 i**	Unpaired Student's t test	N = 2	Medium vs. TNF-α = **<0.0001**<br>Medium vs. INF-γ = **<0.0001**<br>Medium vs. IL-1β = **0.0027**<br>Medium vs. IL-8 = **0.0116**<br>Medium vs. M-CSF = **0.0118**<br>Medium vs. IGF-I = **0.0116**<br>Medium vs. cocktail 1 (TNF-α, INF-γ and IL-1β) = **0.0024**<br>Medium vs. cocktail 2 (IL-8, M-CSF and IGF-I) = **0.0116**
**4 j**	Unpaired Student's t test	N = 2	Medium vs. TNF-α = **0.0010**<br>Medium vs. INF-γ = **0.0018**<br>Medium vs. IL-1β = **0.0222**<br>Medium vs. IL-8 = **0.0027**<br>Medium vs. M-CSF = **0.0237**<br>Medium vs. IGF-I = **<0.0001**<br>Medium vs. cocktail 1 (TNF-α, INF-γ and IL-1β) = **0.0281**<br>Medium vs. cocktail 2 (IL-8, M-CSF and IGF-I) = **0.0011**
**5 b**	Unpaired Student's t test	N = 2	Medium vs. TNF-α = **0.0039**<br>Medium vs. INF-γ = **0.0169**<br>Medium vs. IL-1β = **0.0070**<br>Medium vs. IL-8 = **0.0010**<br>Medium vs. M-CSF = **0.0047**<br>Medium vs. IGF-I = **0.0085**<br>Medium vs. cocktail 1 (TNF-α, INF-γ and IL-1β) = **0.0015**<br>Medium vs. cocktail 2 (IL-8, M-CSF and IGF-I) = **0.0011**<br>Medium vs. Hypoxia 12 h = **0.0040**<br>Medium vs. Hypoxia 18 h = **0.0020**
**5 c**	Unpaired Student's t test	N = 3	Medium vs. TNF-α = **0.0201**<br>Medium vs. INF-γ = **0.0010**<br>Medium vs. IL-1β = **0.0384**<br>Medium vs. IL-8 = **0.0197**<br>Medium vs. M-CSF = **0.0212**<br>Medium vs. IGF-I = **0.0451**

			Medium vs. cocktail 1 (TNF-α, INF-γ and IL-1β) = **0.0137**<br>Medium vs. cocktail 2 (IL-8, M-CSF and IGF-I) = **0.0774**<br>Medium vs. Hypoxia 12 h = **0.0119**<br>Medium vs. Hypoxia 18 h = **0.0367**

2. Supplementary videos

Video S1: Live cell imaging video of HIF-1α-mKate2-expressing cells incubated with cell culture medium (control group).

Video S2: Live cell imaging video of HIF-1α-mKate2-expressing cells treated with 50 ng/ml TNF-α.

Video S3: Live cell imaging video of HIF-1α-mKate2-expressing cells treated with 50 ng/ml INF-γ.

Video S4: Live cell imaging video of HIF-1α-mKate2-expressing cells treated with 50 ng/ml IL-1β.

Video S5: Live cell imaging video of HIF-1α-mKate2-expressing cells treated with 50 ng/ml IL-8.

Video S6: Live cell imaging video of HIF-1α-mKate2-expressing cells treated with 50 ng/ml M-CSF.

Video S7: Live cell imaging video of HIF-1α-mKate2-expressing cells treated with 50 ng/ml IGF-I.

Video S8: Live cell imaging video of HIF-1α-mKate2-expressing cells treated with 50 ng/ml cocktail 1 (TNF-α, INF-γ and IL-1β).

Video S9: Live cell imaging video of HIF-1α-mKate2-expressing cells treated with 50 ng/ml cocktail 2 (IL-8, M-CSF and IGF-I).

All videos consist of four parts (Supplementary Figure 2).

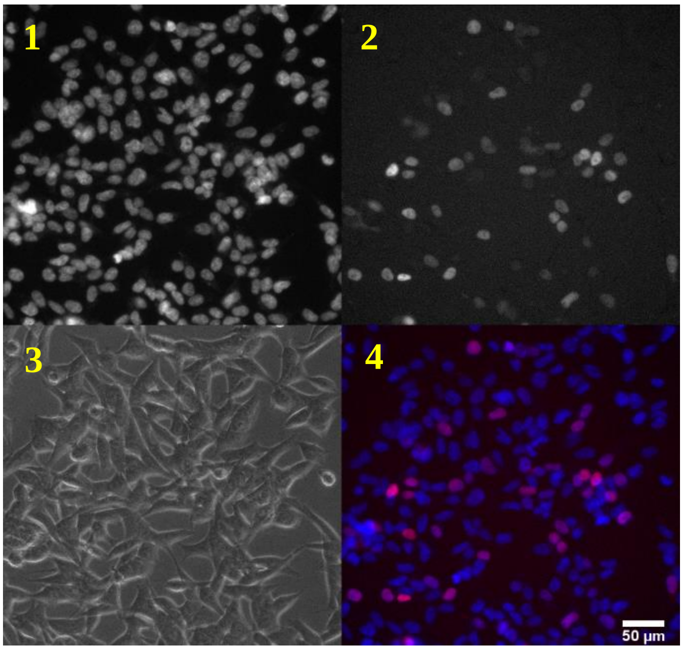

Supplementary Figure S2. Illustration of a selected frame of live cell imaging video. 1: Hoechst 33342 nuclear staining, 2: HIF-1-mKate2 signal, 3: phase contrast, 4: merged Hoechst 33342 and mKate2 signals in the nucleus. MFs caused normoxic HIF-1α stabilization in a time-dependent manner (videos S2-S9).

Resources Table

Table S2. Antibodies

Target antigen	Vendor or Source	Catalog #	Working concentration	Persistent ID/URL
HIF-1α	BD Biosciences	610959	IB: 1:250<br>IF: 1:100	https://www.bdbiosciences.com/en-eu/products/reagents/microscopy-imaging-reagents/immunofluorescence-reagents/purified-mouse-anti-human-hif-1.610958
VEGF	Santa Cruz Biotechnology	sc-7269	IB: 1:500	https://www.scbt.com/p/vegf-antibody-c-1?gclid=EAIaIQobChMI7LabmsPc-QIV0oxoCR1hsQ8tEAAYASAAEgI8FfD_BwE
β-actin	Sigma–Aldrich	A2228	IB: 1:1000	https://www.sigmaaldrich.com/DE/en/substance/monoclonalantibactinantibodyproducedinmouse1234598765?gclid=Cj0KCQjw39uYBhCLARIsAD_SzMRiv5vzbVC8hVkYyscey5mIdE36TmZqfxxs6rBzbHxuHT3Rvd7C8hwaAs_WEALw_wcB
VE-Cadherin	Cell Signaling Technology	#2158	IF: 1:200	https://www.cellsignal.com/products/primary-antibodies/ve-cadherin-antibody/2158?site-search-type=Products&N=4294956287&Ntt=%232158&fromPage=plp&_requestid=2094312
ZO-1	Cell Signaling Technology	#13663	IF: 1:400	https://www.cellsignal.com/products/primary-antibodies/zo-1-d6l1e-rabbit-mab/13663?site-search-type=Products&N=4294956287&Ntt=%2313663&fromPage=plp&_requestid=2094442
Goat IgG anti-Mouse IgG (H+L)-HRPO	Dianova GmbH	115-035-003	IB: 1:5000	https://www.dianova.com/en/shop/115-035-003-goat-igg-anti-mouse-igg-hl-hrpo-minx-none/

Goat anti-Mouse IgG, Alexa Fluor™ 594	Invitrogen, Thermo Fisher Scientific	A-11005	IF: 1:200	https://www.thermofisher.com/antibody/product/Goat-anti-Mouse-IgG-H-L-Cross-Adsorbed-Secondary-Antibody-Polyclonal/A-11005
Goat anti-Rabbit IgG, Alexa Fluor™ 594	Invitrogen, Thermo Fisher Scientific	A-11037	IF: 1:200	https://www.thermofisher.com/antibody/product/Goat-anti-Rabbit-IgG-H-L-Highly-Cross-Adsorbed-Secondary-Antibody-Polyclonal/A-11037
Goat anti-Rabbit IgG, Alexa Fluor™ 488	Invitrogen, Thermo Fisher Scientific	A32731	IF: 1:200	https://www.thermofisher.com/antibody/product/Goat-anti-Rabbit-IgG-H-L-Highly-Cross-Adsorbed-Secondary-Antibody-Polyclonal/A32731

Table S3. Cultured cells

Name	Vendor or Source	Sex (F, M, or unknown)	Persistent ID/URL
Immortalized Human Coronary Artery Endothelial Cells	Applied Biological Material	F	https://www.abmgood.com/immortalized-human-coronary-artery-endothelial-cells.html
HEK293	DSMZ	unknown	https://www.dsmz.de/collection/catalogue/details/culture/ACC-305

Table S4. Other

Description	Source/Repository	Persistent ID/URL
AAVS1 TALE nuclease kit	SBI	https://www.systembio.com/products/crispr-cas9-systems/aavs1-safe-harbor-targeting?cat=155
Bio-Plex Pro Human Cytokine 27-plex Assay	Bio-Rad	https://www.bio-rad.com/de-de/sku/M500KCAF0Y-bio-plex-pro-human-cytokine-27-plex-assay?ID=M500KCAF0Y
Human IGF-I	Peprotech	https://www.peprotech.com/en/recombinant-human-igf-i
Human IL-1β	Miltenyi Biotec	https://www.miltenyibiotec.com/DE-en/products/human-il-1b.html#gref
Human IL-8	Miltenyi Biotec	https://www.miltenyibiotec.com/DE-en/products/human-il-8.html#research-grade:10-ug

Human IFN-γ	Peprotech	https://www.peprotech.com/en/recombinant-human-ifn-2-2
Human M-CSF	Peprotech	https://www.peprotech.com/en/recombinant-human-m-csf
Human TNF-α	R&D Systems	https://www.rndsystems.com/products/recombinant-human-tnf-alpha-hek293-expressed-protein-cf_10291-ta

Original blots used for analysis and graphic representation

Figure S3a:

HIF-1α, 6 h

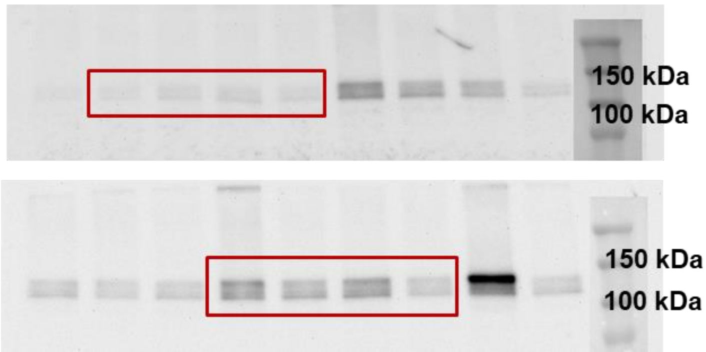

β-actin, 6 h

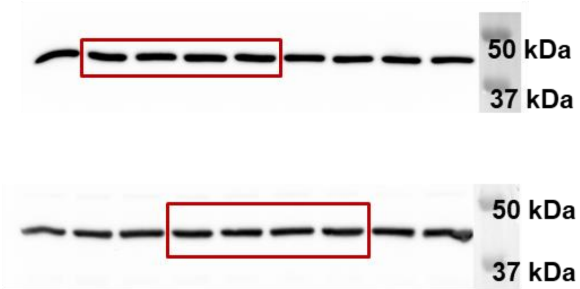

HIF-1α, 12 h

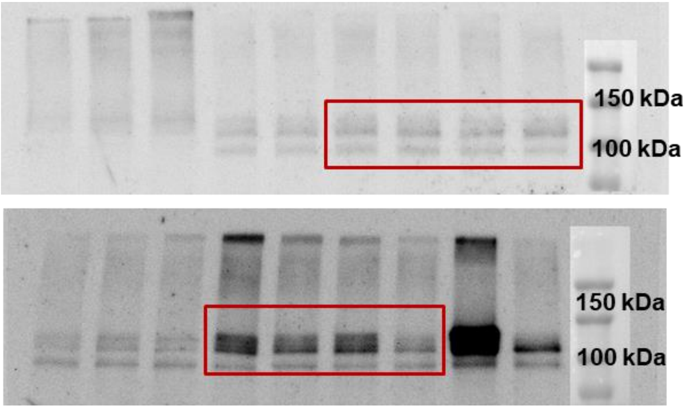

β-actin, 12 h

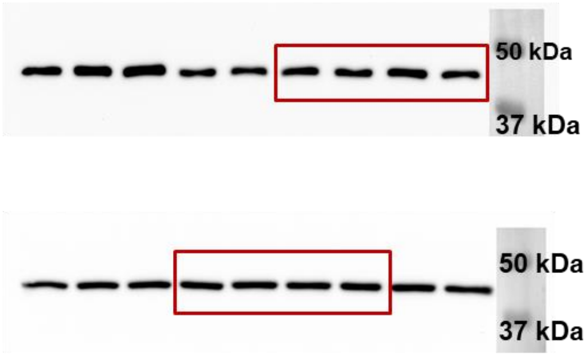

HIF-1α, 18 h

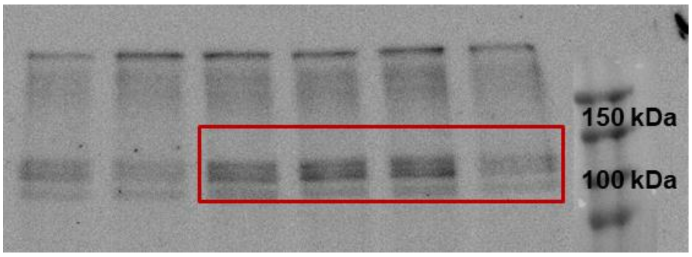

β-actin, 18 h

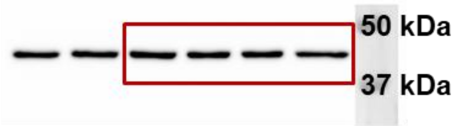

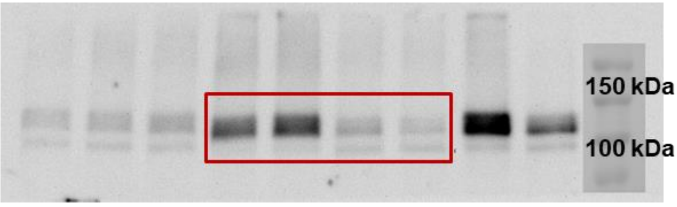

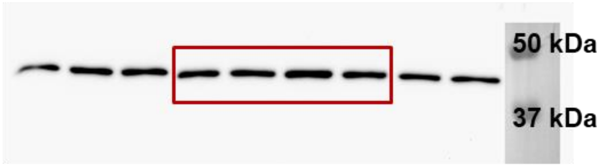

HIF-1α, 24 h

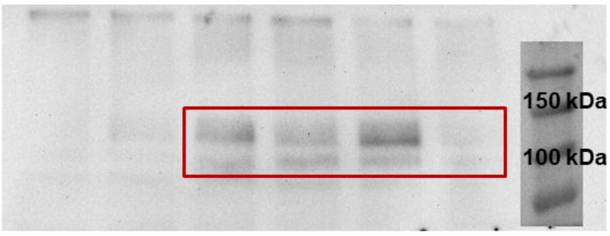

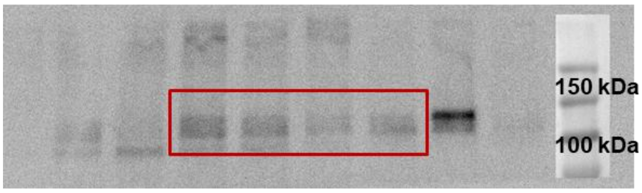

β-actin, 24 h

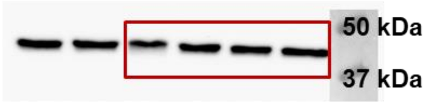

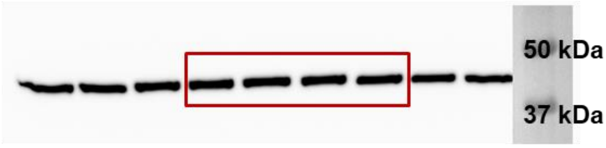

Figure S3b:

HIF-1α

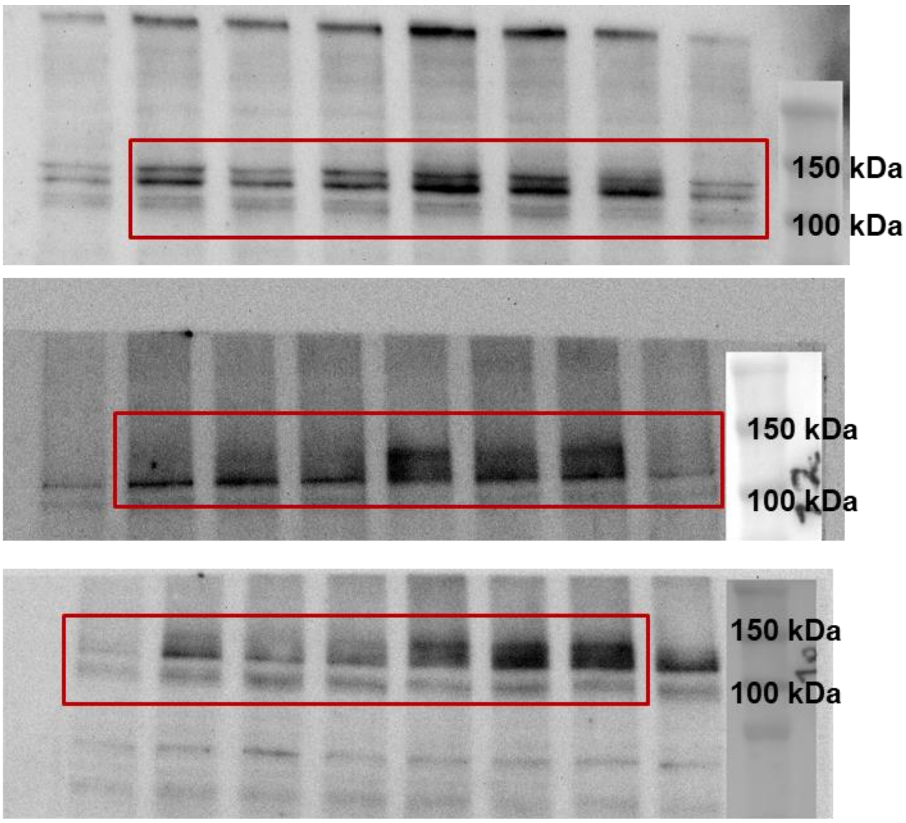

β-actin

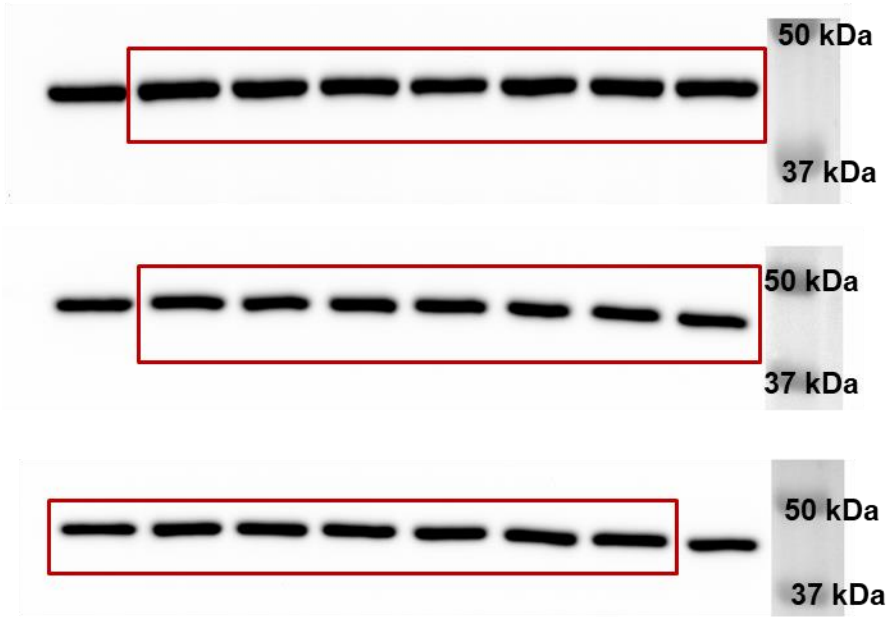

Figure S3c:

HIF-1α

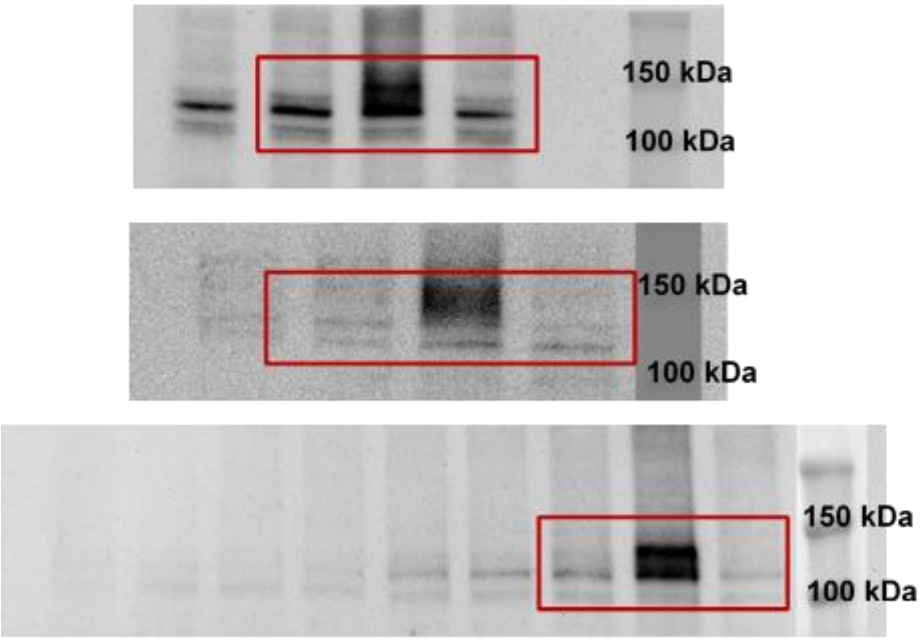

β-actin

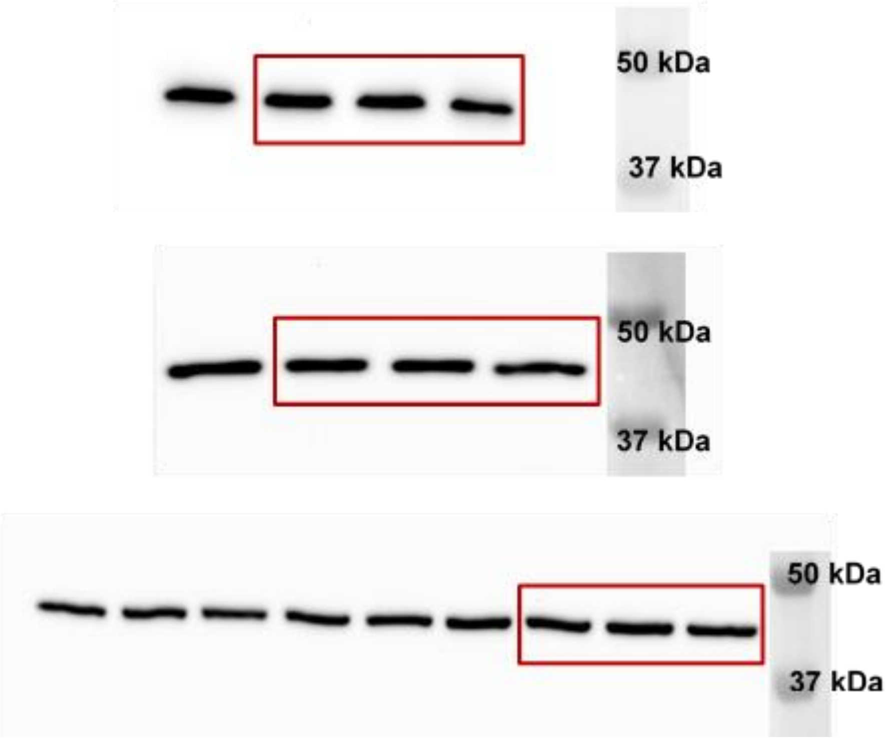

Figure S4b:

VEGF

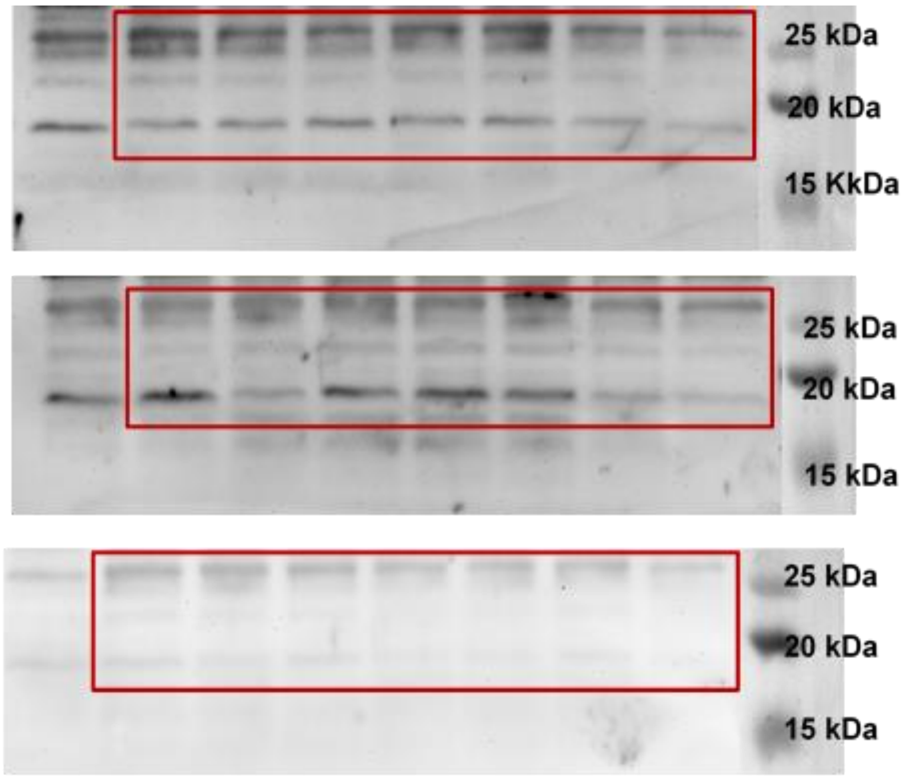

β-actin

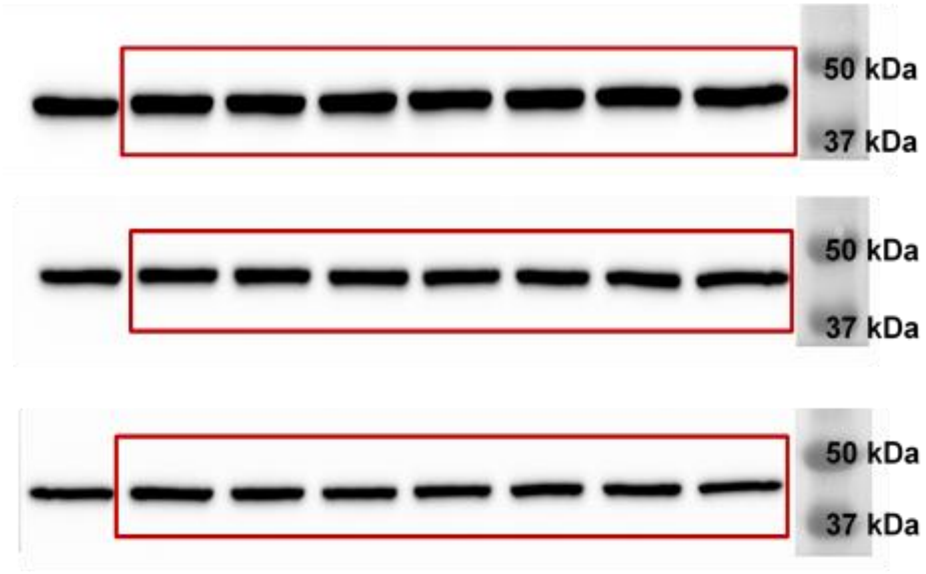

Supplement: Supplementary file 1 [file cells-11-03878-s001.zip › cells-1995865-SI.pdf]
